# National dengue surveillance, Cambodia 2002–2020

**DOI:** 10.2471/BLT.23.289713

**Published:** 2023-07-05

**Authors:** Christina Yek, Yimei Li, Andrea R Pacheco, Chanthap Lon, Veasna Duong, Philippe Dussart, Katherine I Young, Sophana Chea, Sreyngim Lay, Somnang Man, Souv Kimsan, Chea Huch, Rithea Leang, Rekol Huy, Cara E Brook, Jessica E Manning

**Affiliations:** a Laboratory of Malaria and Vector Research, National Institute of Allergy and Infectious Diseases, 12735 Twinbrook Parkway, Rockville, MD 20852, United States of America (USA).; b Department of Ecology and Evolution, University of Chicago, Chicago, USA.; c International Center of Excellence in Research, National Institute of Allergy and Infectious Diseases, Phnom Penh, Cambodia.; d Virology Unit, Institut Pasteur du Cambodge, Phnom Penh, Cambodia.; e Biological Sciences Department, University of Texas at El Paso, El Paso, USA.; f National Center of Parasitology, Entomology, and Malaria Control, Ministry of Health, Phnom Penh, Cambodia.

## Abstract

Global dengue incidence has increased dramatically over the past few decades from approximately 500 000 reported cases in 2000 to over 5 million in 2019. This trend has been attributed to population growth in endemic areas, rapid unplanned urbanization, increasing global connectivity, and climate change expanding the geographic range of the *Aedes spp*. mosquito, among other factors. Reporting dengue surveillance data is key to understanding the scale of the problem, identifying important changes in the landscape of disease, and developing policies for clinical management, vector control and vaccine rollout. However, surveillance practices are not standardized, and data may be difficult to interpret particularly in low- and middle-income countries with fragmented health-care systems. The latest national dengue surveillance data for Cambodia was published in 2010. Since its publication, the country experienced marked changes in health policies, population demographics, climate and urbanization. How these changes affected dengue control remains unknown. In this article, we summarize two decades of policy changes, published literature, country statistics, and dengue case data collected by the Cambodia National Dengue Control Programme to: (i) identify important changes in the disease landscape; and (ii) derive lessons to inform future surveillance and disease control strategies. We report that while dengue case morbidity and mortality rates in Cambodia fell between 2002 and 2020, dengue incidence doubled and age at infection increased. Future national surveillance, disease prevention and treatment, and vector control policies will have to account for these changes to optimize disease control.

## Résumé

Le taux d'incidence de la dengue dans le monde a considérablement augmenté au cours des dernières décennies, passant d'environ 500 000 cas notifiés en 2000 à plus de 5 millions en 2019. Cette tendance est attribuée à la croissance démographique dans les zones d'endémie, à l'urbanisation rapide non planifiée, au développement de la connectivité à l'échelle internationale, ainsi qu'au changement climatique, qui agrandit le territoire géographique du moustique *Aedes spp*., entre autres. La communication des données de surveillance de la dengue est essentielle pour comprendre l'étendue du problème, identifier les principales variations de contexte entourant la maladie et mettre au point des politiques pour la prise en charge clinique, la lutte contre les vecteurs et le déploiement des vaccins. Les pratiques en matière de surveillance ne sont toutefois pas standardisées et les données peuvent être difficiles à interpréter, surtout dans les pays à revenu faible et intermédiaire où les systèmes de soins de santé sont fragmentés. Les données de surveillance les plus récentes concernant la dengue au Cambodge ont été publiées en 2010. Depuis leur publication, le pays a subi de profondes mutations au niveau des politiques de santé, de l'évolution démographique, du climat et de l'urbanisation. L'impact de ces mutations sur la lutte contre la dengue reste à établir. Dans le présent article, nous résumons deux décennies d'amendements politiques, de documentation, de statistiques nationales et d'informations collectées sur les cas par le programme cambodgien de lutte contre la dengue afin de: (i) définir les changements importants survenus dans le contexte entourant la maladie; mais aussi (ii) tirer des leçons en vue d'élaborer, à l'avenir, des stratégies de surveillance et de lutte contre la maladie. Nous signalons qu'en dépit d'une baisse des taux de morbidité et de mortalité liés aux cas de dengue entre 2002 et 2020 au Cambodge, son incidence a doublé et l'âge des patients au moment de l'infection a augmenté. Les futures politiques nationales de surveillance, de prévention et de traitement de la dengue, mais aussi de lutte contre ses vecteurs, devront tenir compte de ces changements de façon à mieux maîtriser la maladie.

## Resumen

La incidencia del dengue a nivel mundial ha aumentado considerablemente en las últimas décadas, desde aproximadamente 500 000 casos notificados en el año 2000 a más de 5 millones en 2019. Esta tendencia se ha atribuido al crecimiento de la población en zonas endémicas, a una urbanización rápida y no planificada, al aumento de la conectividad a nivel mundial y al cambio climático, que está permitiendo una distribución geográfica más amplia del mosquito *Aedes spp*., entre otros factores. Para comprender la magnitud del problema resulta clave la notificación de datos sobre vigilancia del dengue, la identificación de cambios importantes dentro del escenario de la enfermedad, la creación de políticas enfocadas a la gestión clínica, así como el control de vectores y la implantación de la vacuna. Sin embargo, las prácticas sobre vigilancia no están estandarizadas y es posible que sea difícil interpretar los datos, especialmente en países con ingresos medios y bajos, que cuentan con sistemas fragmentados de atención sanitaria. Los datos nacionales más recientes sobre vigilancia del dengue en Camboya se publicaron en 2010. Desde su publicación, el país experimentó cambios significativos en las políticas sanitarias, la demografía de la población, el clima y la urbanización. Aún no se sabe cómo afectaron dichos cambios al control del dengue. En el presente artículo, resumimos dos décadas de cambios políticos, de bibliografía publicada, de datos estadísticos a nivel nacional y datos sobre casos de dengue recopilados por el programa nacional de control de dengue en Camboya, con el fin de: (i) identificar cambios importantes en el escenario de la enfermedad; y (ii) extraer conclusiones para orientar futuras estrategias sobre vigilancia y control de la enfermedad. Informamos de que, aunque las tasas de morbilidad y mortalidad de los casos de dengue en Camboya descendieron entre 2002 y 2020, la incidencia del dengue se duplicó y la edad de infección aumentó. Las futuras políticas nacionales sobre vigilancia, prevención y tratamiento de la enfermedad y control de vectores deberán tener en cuenta estos cambios para optimizar el control de la enfermedad.

## ملخص

زاد معدل الإصابة بحمى الضنك على مستوى العالم بشكل كبير خلال العقود القليلة الماضية، من حوالي 500000 حالة تم الإبلاغ عنها في عام 2000 إلى ما يزيد عن 5 ملايين حالة في عام 2019. ويعزى هذا الاتجاه إلى النمو السكاني في المناطق الموبوءة، والتحضر السريع غير المخطط له، وزيادة الاتصال العالمي، وتغير المناخ الذي يمتد ليشمل النطاق الجغرافي لبعوضة *Aedes spp *، إلى جانب عوامل أخرى. يعد الإبلاغ عن بيانات رصد حمى الضنك أمرًا أساسيًا لفهم حجم المشكلة، وتحديد التغييرات المهمة في واقع المرض، ووضع سياسات للإدارة الإكلينيكية، ومكافحة النواقل، وطرح اللقاح. ومع ذلك، فإن ممارسات الرصد ليست قياسية وقد يكون من الصعب تفسير البيانات خاصة في الدول ذات الدخل المنخفض والمتوسط الدخل، ذات أنظمة الرعاية الصحية المتدهورة. تم نشر أحدث بيانات الرصد الوطني لحمى الضنك في كمبوديا في عام 2010. ومنذ نشرها، شهدت البلاد تغيرات ملحوظة في السياسات الصحية، والتركيبة السكانية، والمناخ، والتوسع الحضري. إن كيفية تأثير هذه التغييرات على مكافحة حمى الضنك يظل غير معروف. نلخص في هذا المقال عقدين من التغييرات في السياسة، والأدبيات المنشورة، وإحصاءات الدولة، وبيانات حالة حمى الضنك التي جمعها برنامج مكافحة حمى الضنك الوطني في كمبوديا من أجل: (1) تحديد التغييرات المهمة في واقع المرض؛ و(2) استخلاص الدروس لتوجيه استراتيجيات رصد ومكافحة المرض في المستقبل. لقد أعلننا أنه في حين انخفضت معدلات الإصابة بالمرض ونسبة الوفيات بسبب حمى الضنك في كمبوديا بين عامي 2002 و2020، فقد تضاعف معدل الإصابة بحمى الضنك، وزاد العمر عند الإصابة. سيكون على الرصد الوطني في المستقبل، والوقاية من المرض وعلاجه، وسياسات مكافحة ناقلات الأمراض أن تأخذ في الاعتبار هذه التغييرات لتحسين مكافحة المرض.

## 摘要

在过去的几十年里，全球登革热发病率急剧增加，从 2000 年的约 50 万例报告病例增至 2019 年的 500 多万例。出现该趋势的原因在于疾病流行区域人口增长、无规划地加快城市化进程、全球连通性增强以及气候变化导致伊蚊的地理分布范围扩大等等。报告登革热监测数据是了解问题严重程度、确定疾病情形相关重要变化以及制定临床管理、病媒控制和疫苗推广相关政策的关键。但是，监测工作无法实现标准化，且数据还有可能难以分析，特别是在医疗体系较为分散的中低收入国家。柬埔寨的最新全国登革热监测数据公布于 2010 年。自数据公布以来，该国的卫生政策、人口统计、气候和城市化方面均发生了显著变化。这些变化会对控制登革热产生何种影响仍然未知。在本文中，我们总结了 20 年来的政策变化、已发表文献、国家统计数据以及根据柬埔寨国家登革热控制计划收集的登革热病例数据，旨在：（i）确定疾病情形相关重要变化；以及（ii）汲取经验教训，为未来的监测工作和疾病控制战略提供实用信息。我们的报告指出，虽然在 2002-2020 年间，柬埔寨登革热病例的发病率和死亡率均有所下降，但登革热患病率翻了一番，且感染者的年龄范围扩大。未来的国家监测、疾病预防和治疗以及病媒控制相关政策均需考虑这些变化，以优化疾病控制。

## Резюме

За последние несколько десятилетий заболеваемость денге в мире резко возросла: с примерно 500 000 зарегистрированных случаев в 2000 году до более 5 миллионов в 2019 году. Эта тенденция объясняется среди прочих факторов ростом населения на эндемичных территориях, быстрой незапланированной урбанизацией, растущей глобальной связью, а также изменением климата, расширяющим географический ареал комара *Aedes spp*. Отчетность о данных эпиднадзора за денге имеет ключевое значение для понимания масштабов проблемы, выявления важных изменений в характере заболевания и разработки политики клинического лечения, борьбы с переносчиками и внедрения вакцин. Однако практика осуществления эпиднадзора не стандартизирована, и интерпретация данных может быть затруднена, особенно в странах с низким и средним уровнем доходов и фрагментированными системами здравоохранения. Последние данные эпиднадзора за денге на национальном уровне в Камбодже были опубликованы в 2010 году. Со времени этой публикации в стране произошли заметные изменения в политике здравоохранения, демографических характеристиках населения, климате и урбанизации. Как эти изменения повлияли на борьбу с денге, остается неизвестным. В этой статье подведены итоги двух десятилетий изменений в политике, опубликованной литературы, статистики по стране и данных о случаях денге, собранных в рамках национальной программы по борьбе с денге в Камбодже, чтобы: (i) выявить важные изменения в характере заболевания; и (ii) извлечь уроки для информирования будущих стратегий эпиднадзора и контроля заболевания. В период с 2002 по 2020 год заболеваемость и смертность от денге в Камбодже снизились, однако область распространения выросла в два раза и возраст инфицированных увеличился. В будущем эпиднадзор на национальном уровне, профилактика и лечение заболеваний, а также политика борьбы с переносчиками должны будут учитывать эти изменения в целях оптимизации борьбы с распространением заболеваний.

## Introduction

The global incidence of reported dengue has increased by ten-fold over the last two decades, from 505 430 cases in 2000 to 5.2 million in 2019.^1^ To contain infection and manage clinical disease, accurate estimates of dengue burden are needed to ensure appropriate allocation of resources, especially during rollouts of the novel tetravalent vaccines in dengue-endemic areas.^2^^,^^3^ Nevertheless, countries with the highest dengue prevalence predominantly rely on resource-scarce national surveillance systems that are often based on case identification by clinical presentation of symptoms and passive reporting, leading to large underestimation of disease burden.^4^

Cambodia is a lower-middle income country located in a belt of dengue-endemic countries in Asia that together contribute to an estimated 70% of global dengue cases.^1^ National dengue surveillance data for Cambodia was last published in 2010^5^ and covered a period of dynamic change from 1980 to 2008, during which post-civil war improvements in public health infrastructure led to improved systems for surveillance and vector control. Enhanced surveillance was introduced in 2001; initial analysis of complete case data from 2002–2008 found no clear trends in dengue incidence, and uncertain impact of larvicide distribution. Since 2008, changes in health policies, population demographics, and land use have occurred alongside rapid industrialization in the country. Furthermore, rising global temperatures attributed to climate change have enhanced growth conditions for the vector for dengue, the *Aedes spp.* mosquito.^6^ How these changes affect dengue control in Cambodia remains unknown.

Here we summarize two decades of policy changes, published literature, country statistics, and data from the Cambodian National Dengue Control Programme to identify important changes in the disease landscape, and derive lessons to inform future surveillance and disease control strategies.

## Methods

### Data sources

National dengue surveillance data, including de-identified individual case data for both dengue and chikungunya cases and viral serotyping, are available upon request from the Cambodian health ministry. Monthly dengue incidence is also accessible via WHO weekly reports. We obtained census data from the 1998, 2008, and 2019 national Cambodian population censuses.^7^ We computed population growth rate using the exponential growth formula from the World Bank^8^ to extrapolate data for intercensus years. We obtained climate data as daily aggregates (that is, total precipitation and mean 2m air temperature) from Google’s Earth Engine,^9^ and land use data (that is, forested area and urban population) from the World Bank.^10^ To identify published literature on dengue in Cambodia, we performed a systematic search using the search terms “dengue” and “Cambodia” in MEDLINE® and Embase® databases without any language or time restriction. We updated the search on 22 May 2023.

### Dengue and climate models

To examine whether dengue case incidence, outcome, phenotype, and age of infection changed between 2002 and 2020,we fitted generalized linear models to time series of the response variables of (i) monthly dengue case numbers; (ii) case fatality rates; (iii) case proportions of dengue haemorrhagic fever; (iv) case proportions of dengue shock syndrome: and (v) mean age of infected individuals derived from national surveillance data, using year, monthly average temperature, and monthly total precipitation at the national level as fixed predictor effects. We fitted models in the Poisson family to crude case numbers and the Gaussian family to all other response variables. Fixed predictor climate variables (temperature and precipitation) were lagged to precede case response data. To identify the most appropriate period for the climate lags, we first optimized a cross-correlation function, testing associations between lag periods spanning up to one year for mean monthly temperature and total precipitation as compared with the response variable of monthly case counts. We calculated optimal lags of −3 and −11 months between dengue cases, and temperature and precipitation, respectively; climate variables were subsequently lagged by these durations before inclusion in generalized linear models. To examine whether climate variables could be driving inter-annual changes as well, we next explored whether mean monthly temperature and total precipitation (as significant predictors of dengue case variables) changed significantly over the examined period. To do this, we fitted generalized additive models to time series of monthly climate data, including a fixed predictor of year, and controlling for intra-annual variability via incorporation of a monthly smoothing term, with the number of smoothing knots fixed at 7, and incorporating a cyclic cubic smoothing spline.

## Surveillance and management

### National surveillance

Since 2001, the Cambodia National Dengue Control Programme has collected demographic and clinical data on hospitalized dengue cases at government-supported health-care facilities across all 25 provinces.^5^ Contributing facilities report clinically diagnosed dengue cases using a standardized case report form that collates de-identified patient data including patient age; sex; home province; hospital admission date; clinical diagnosis (dengue fever, dengue haemorrhagic fever or dengue shock syndrome); and disease outcome (death or survival to discharge). Each month, facilities submit these forms to the dengue control programme and data are stored in a central electronic database. In a minority of patients, facilities perform serologic confirmatory testing at point-of-care for diagnosis, using the SD BIOLINE Dengue Duo rapid test (Abbott®, Chicago, United States of America). The testing is dependent on patient ability to afford the test and availability of test kits. 

In sentinel sites (4 provinces in 2001, with expansion to 15 provinces by 2021), the National Dengue Control Programme performs centralized virologic surveillance using real-time polymerase chain reaction (RT–PCR) for identification of viral serotypes in a subset of samples. 

**Fig. 1:**
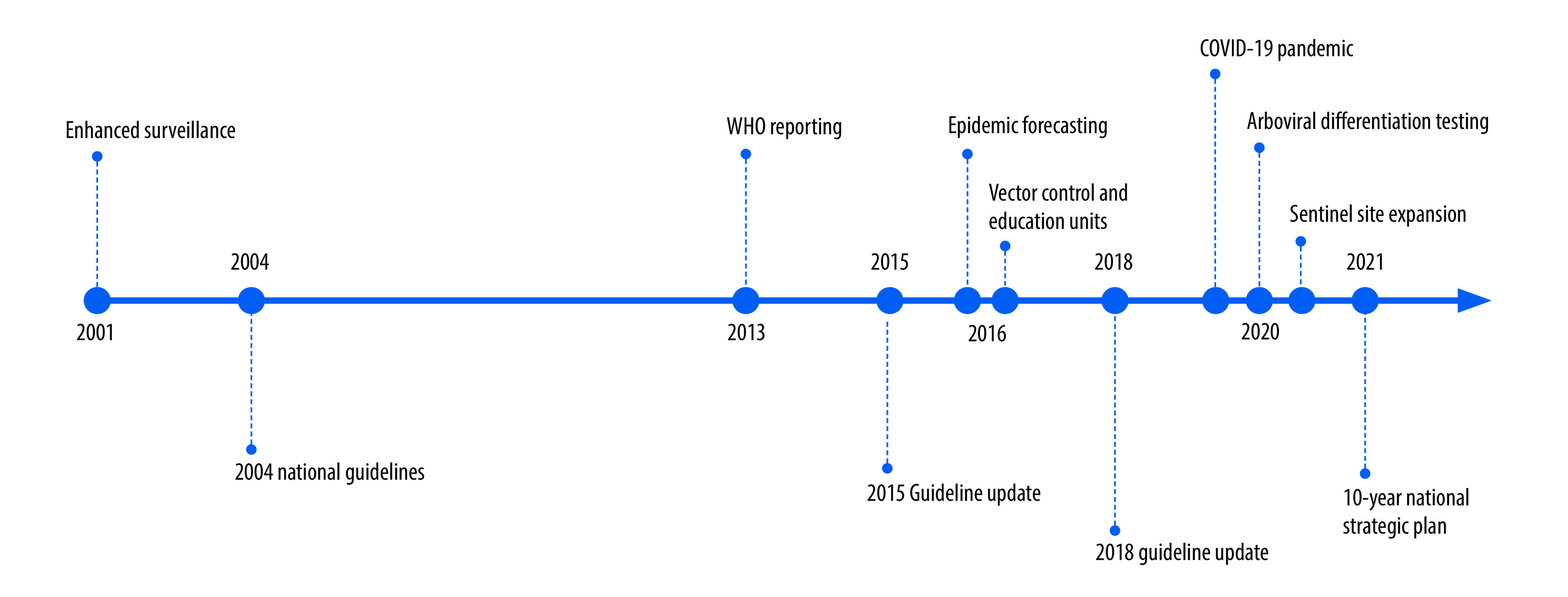
Timeline of major events affecting dengue surveillance and management, Cambodia, 2001–2021

COVID-19: coronavirus disease 2019; WHO: World Health Organization.

Several additional notable events over the past two decades have changed the structure of dengue surveillance and response in Cambodia, including routine contribution to the World Health Organization’s (WHO) dengue situation updates beginning in 2013; introduction of real-time epidemic forecasting and establishment of dedicated vector control units in 2016; and arboviral differentiation testing and expansion of sentinel sites in 2020 (Box 1 and Fig. 1).^11^^–^^13^

Box 1 **Major events affecting dengue surveillance and management, Cambodia, 2001–2021**

***2001 – Enhanced national surveillance***

Standardization of dengue surveillance using WHO clinical case definitions, standardized case report forms and data entry into an electronic database across 25 provinces. Introduction of virologic surveillance in four provinces.

***2004 – National dengue guidelines published***

Guidelines describe diagnosis of dengue fever, haemorrhagic fever, and shock syndrome, along with monitoring and supportive care strategies.

***2013 – Dengue reporting to WHO***

Routine submission of dengue surveillance data for collation as part of WHO’s monthly dengue situation updates.

***2015 – National dengue guidelines updated***

Updated guidelines recommended centralized care at referral centres, and judicious fluid resuscitation of patients; and provided separate guidance for patients with complications of disease and/or higher baseline risk.

***2016 – Epidemic forecasting introduced***

The National Dengue Control Programme introduced an epidemic prediction algorithm to identify early rises in case numbers beyond historic baselines that could signal an impending epidemic with 2–3 months’ lead time. This algorithm was linked to a response system that deployed enhanced vector control and targeted education.

***2016 – Vector control and dengue education units established***

Establishment of dedicated response teams in the National Dengue Control Programme to coordinate vector control efforts and disseminate information on dengue recognition and management to public and clinical sectors.

***2018 – National dengue guidelines updated***

Updated guidelines included additional caps on fluid resuscitation, guidance for older populations, empiric and/or advanced treatment options for complicated disease, and hospital outbreak preparedness planning and response strategies.

***2020 – COVID-19 pandemic***

COVID-19 led to changes in dengue transmission and detection related to societal mobility restrictions, reduced care-seeking behaviour, and disruption in local and national surveillance and vector control activities.

***2020 – Arboviral differentiation testing***

The worldwide Zika virus epidemic in 2016 and a large chikungunya virus outbreak in Cambodia in 2020 led to routine testing of virologic surveillance samples for chikungunya, dengue, and Zika viruses using PCR.

***2020–2021 – More virologic surveillance sites***

Sentinel surveillance sites in 11 provinces were added to original sites in 4 provinces between 2020 and 2021; virologic surveillance now spans 15 of 25 provinces.

***2021 – National strategic plan published***

In consultation with WHO, the National Dengue Control Programme created a 10-year plan for sustainable prevention and control of dengue and other *Aedes* *spp.-*transmitted arboviral diseases.

COVID-19: coronavirus disease 2019; PCR: polymerase chain reaction; WHO: World Health Organization.

### Vector control

In Cambodia, biannual applications of larvicide (temephos) from April to July and August to October occur in tandem with public education campaigns reinforcing the importance of environmental and mechanical control, such as clearing stagnant water and using jar covers on open containers.^5^ Both vector control and public and clinician education have been centrally coordinated by the National Dengue Control Programme since 2001, although dedicated vector control units were only created in 2016 and linked to a new system for epidemic forecasting.^14^ Larvicide application remains highly variable with fluctuation based on resource availability and impact remains uncertain largely due to the presence of informal breeding sites.^5^ More recently, increasing *Aedes* resistance to temephos^15^ prompted evaluation of other insecticides, such as *Bacillus thuringiensis israelensis* and pyriproxyfen, biological control with larvivorous guppy fish, and mechanical control with mosquito traps or covers for open water containers. Multisectoral involvement has supported community-specific mobilization.^16^^,^^17^ However, while potentially efficacious and affordable, these efforts remain largely siloed and exploratory.^18^^–^^20^

### Dengue treatment guidelines

While curative therapies for dengue remain elusive, major advances in the understanding of disease pathophysiology^21^ have contributed to the development of enhanced supportive strategies and improved outcomes over the last two decades. Specifically, studies on fluid resuscitation in critically ill paediatric populations with dengue^22^^–^^24^ and other shock conditions^25^ brought attention to the importance of thoughtful fluid selection and infusion rates to minimize iatrogenic harm. Although data were available from the late 1990s, updates to WHO guidelines were only made in 2009,^26^ while update of the Cambodian national guidelines happened in 2015. The 2015 Cambodian *Dengue guidelines*,^27^ updated from the original 2004 document, introduced several important changes in disease management, including (i) use of the more clinically relevant WHO 2009 disease classification;^28^ (ii) centralized care at provincial referral hospitals, away from variably equipped health posts and unregulated private sector clinics; (iii) limits to fluid resuscitation to avoid masking of haemoconcentration as an early warning sign of dengue haemorrhagic or dengue shock syndrome, and iatrogenic harm including cardiovascular overload; (iv) specific clinical parameters to guide care of younger children, such as age-appropriate blood pressure and haematocrit ranges and decreased fluid rate for infants; (v) algorithmic testing for complications of disease, such as acidosis, bleeding, hypocalcaemia and hypoglycaemia; (vi) treatment plans for infrequent complications, such as hyponatraemia and hepatic encephalopathy; and (vii) recognition of certain high-risk populations, including those with hemoglobinopathies or congenital heart disease, that warrant immediate transfer to a higher level of care.

The 2018 guidelines updates^29^ incorporated additional caps on fluid resuscitation, including emphasis on early fluid discontinuation, a suggested 24-hour cap on colloid volume, and adult-specific infusion rates. The update recommended identification of risk profiles specific to adult populations, including pregnancy and diabetes, requiring heightened concern for clinical deterioration. The 2018 update also recommended empiric treatment of disease complications to avoid delays in care, such as administration of oxygen, calcium gluconate and vitamin K as part of upfront management of dengue haemorrhagic fever or dengue shock syndrome or unresponsive to initial fluid resuscitation. The use of advanced organ support therapies, such as renal replacement and mechanical ventilation was added, although in reality these interventions are limited to major hospitals in Phnom Penh. Finally, the guidelines now include provisions for outbreak preparedness, such as appropriate hospital response plans, and guidelines for inter-hospital acuity-related and/or load-balancing transfers. While laudable, these provisions remain limited by lack of available and properly fitted ambulances which, when combined with exorbitant costs of medical transportation, create challenging logistics for patient access to tertiary care.^30^

## Evolving disease landscape

### Incidence and disease severity

**Fig. 2:**
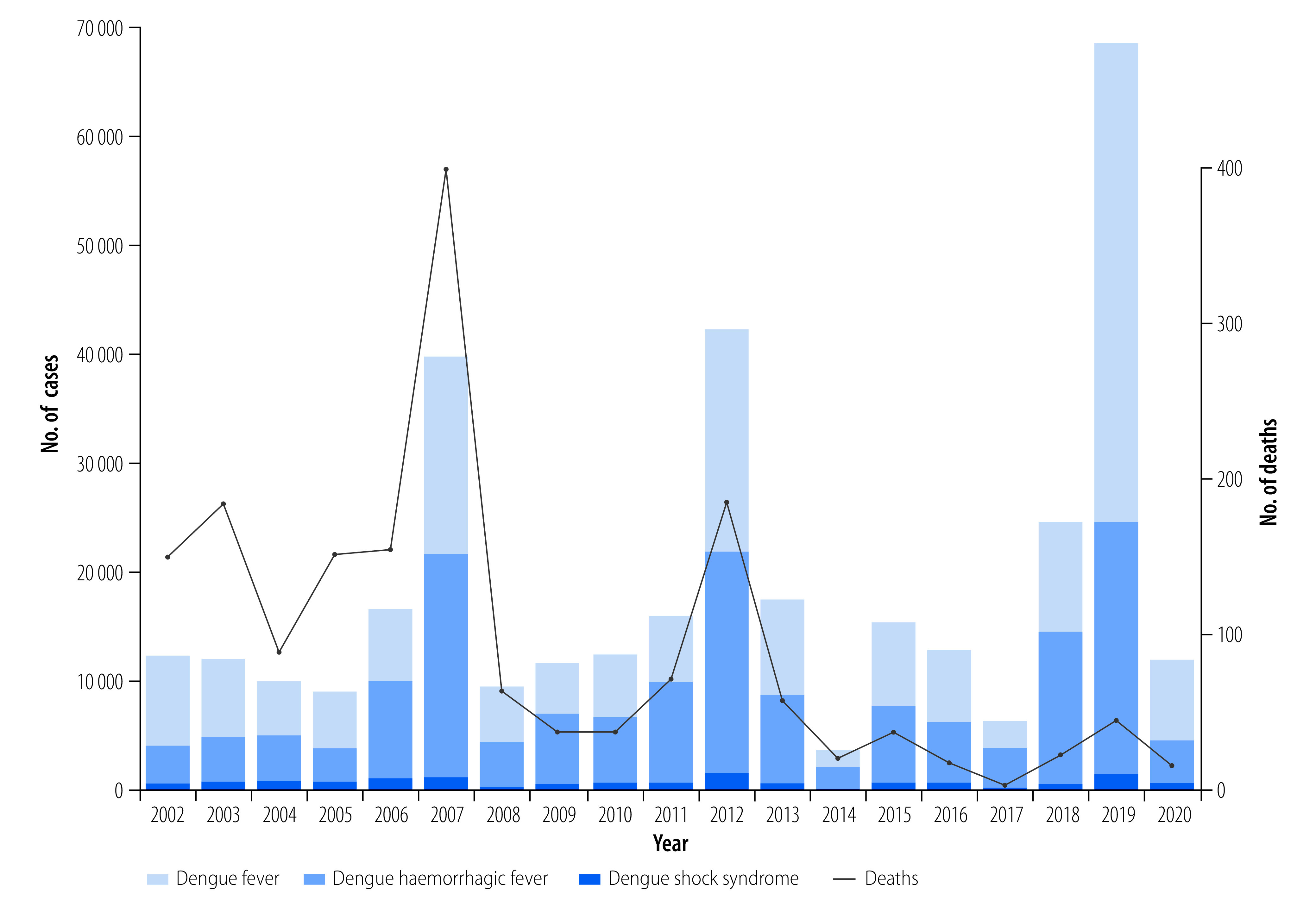
Dengue cases and deaths, Cambodia, 2002–2020

**Fig. 3:**
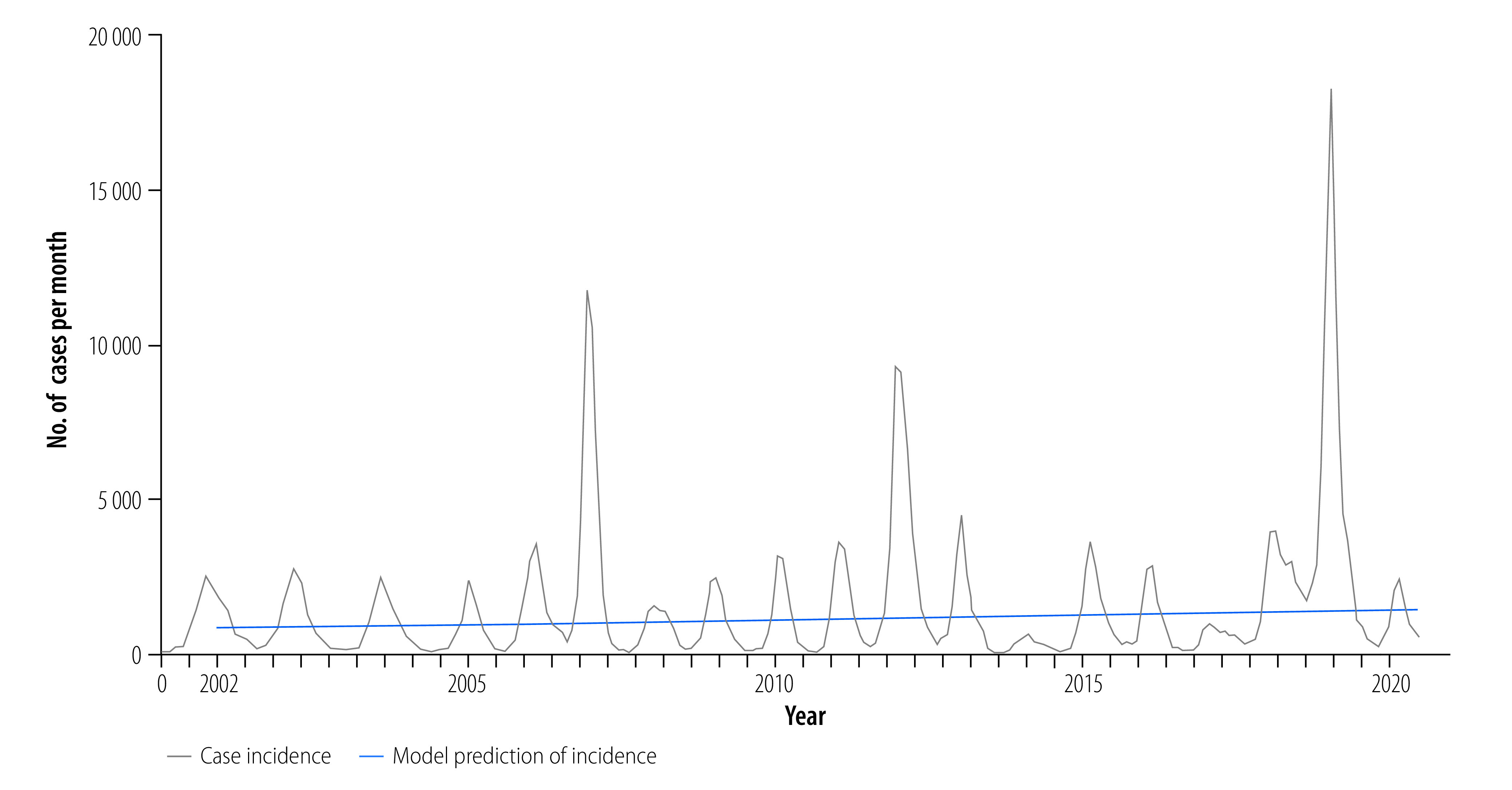
Annual incidence of dengue, Cambodia, 2002–2020

Note: The blue line presents predictions of a generalized linear model fitted to dengue cases, excluding the effects of climate predictors (temperature and precipitation), which control intra-annual variation.

From 2002 to 2020, a total of 353 270 dengue cases were reported to the National Dengue Control Programme (Fig. 2; further information available in the online repository).^31^ Average age-adjusted incidence was 1.75 cases per 1000 persons per year, and ranged as high as 6.27 cases per 1000 persons per year during the large 2019 epidemic. The majority of cases were dengue fever (51%; 180 914/353 270), with dengue haemorrhagic fever and dengue shock syndrome representing 45% (158 536/353 270) and 4% (13 820/353 270) of cases, respectively. Average annual case fatality rate was 0.57% (standard deviation: 0.48). Generalized linear models adjusted for climate factors and fitted to dengue cases from 2002 to 2020 (online repository)^31^ demonstrated a significant increase in annual cases (slope: 0.030; stander error, SE: 0.000334; *P*-value: < 0.001) representing a 2.1-fold increase in dengue incidence over the 19-year period (Fig. 3). Case fatality rates decreased over time (case fatality rates: 1.19% between 2002 and 2007 to 0.19% between 2014 and 2020; slope: −0.17; SE: 0.0056; *P*-value: < 0.001). Similar trends were seen with cases of severe dengue: dengue haemorrhagic fever: 44.26% between 2002 and 2007 to 41.66% between 2014 and 2020 (slope: −0.015; SE: 0.00068; *P*-value: < 0.001), and dengue shock syndrome: 5.43% between 2002 and 2007 to 2.73% between 2014 and 2020 (slope: −0.067; SE: 0.0018; *P*-value: < 0.001); online repository).^31^

### Active surveillance

Between 2002 and 2020, four studies reported results of active surveillance of dengue cases in Cambodia.^32^^–^^35^ Three studies reported results of population-based febrile surveillance in central Cambodia (Kampong Cham province) between 2006 and 2008, in which dengue incidence was estimated at 13.4–57.8 per 1000 person per season with variation by age group and locality.^32^^–^^34^ Two of these publications estimated burden of additional dengue cases 3.9- to 29.0-fold higher than that captured by national surveillance (1.1–5.7 per 1000 person per season).^32^^,^^34^ Between 2018 to 2020, a longitudinal cohort captured clinically apparent dengue in children aged 2 to 9 years in Kampong Speu province,^35^ with case incidence 5.0-fold that detected by national surveillance among children in the same age group and province, 3.0 versus 0.6 cases per 1000 person-months, respectively. Of note, these estimates of underreporting are limited by heterogeneous methods, non-random selection of cohorts of interest, small sample sizes, and use of different clinicopathologic criteria to define dengue cases. Similar limitations exist for empirical studies on underreporting published elsewhere,^4^ and data should be interpreted with caution; ultimately, nationally representative longitudinal cohort studies with careful selection of case definitions are needed to better understand degree of incomplete case capture by national passive surveillance, but such studies are resource-intensive and may be impractical in the long term.

### Age structure of cases

**Fig. 4:**
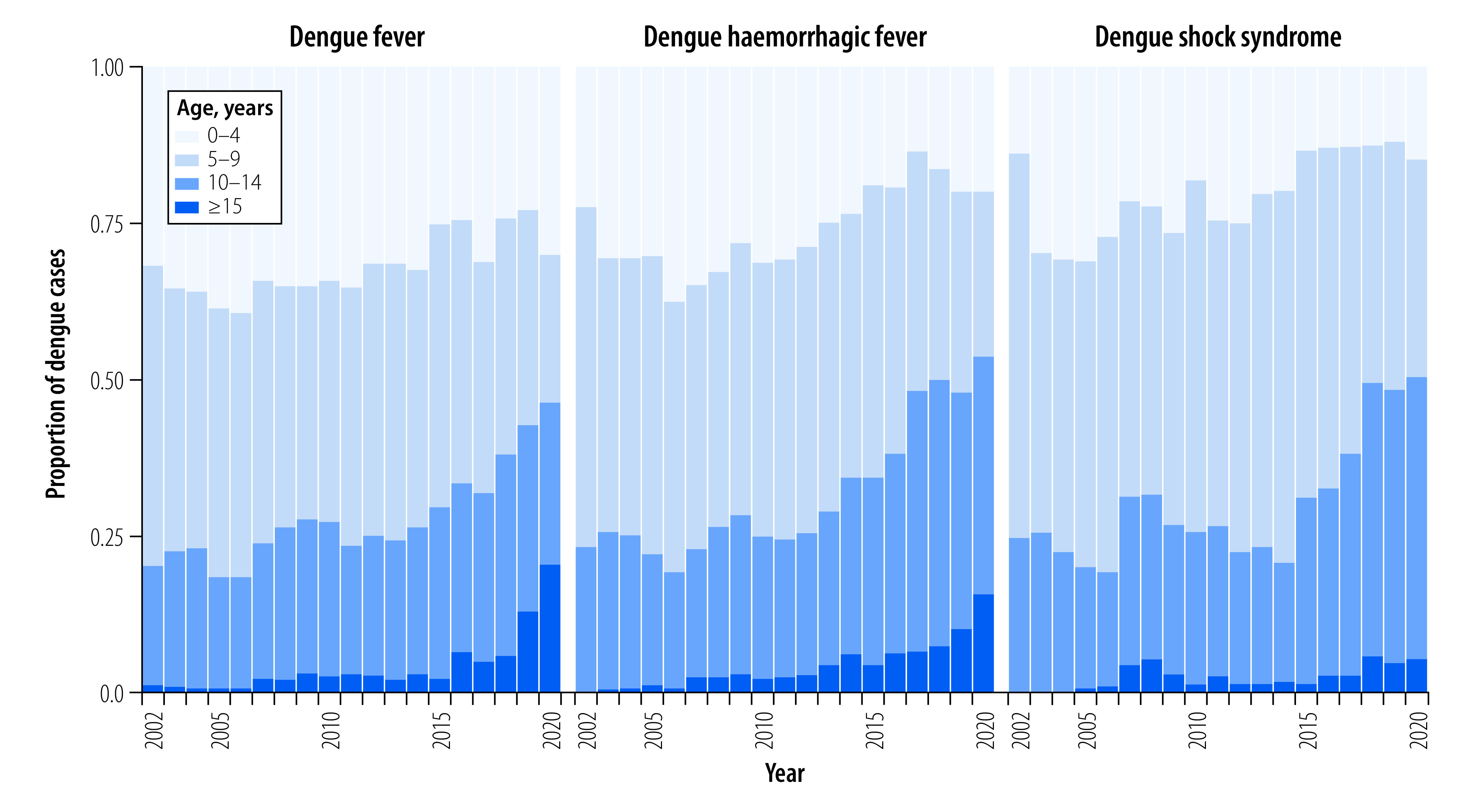
Dengue cases stratified by clinical diagnosis, Cambodia, 2002–2020

**Fig. 5:**
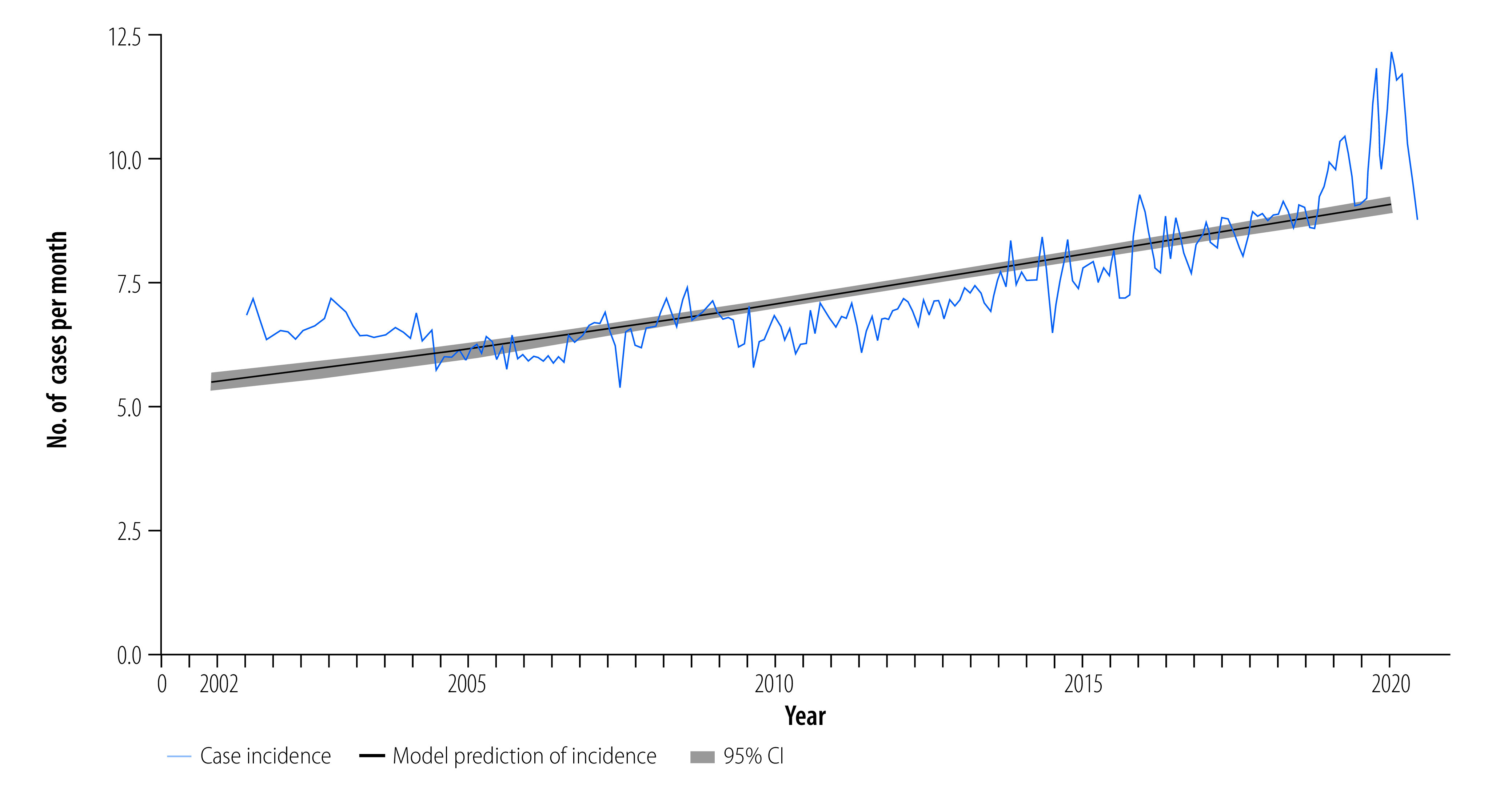
Mean age of dengue-infected individuals, Cambodia, 2002–2020

Before 2014, children aged 5 to 9 years represented the majority of dengue cases, followed by children aged 0 to 4 years. From 2014 to 2019, children aged 10 to 14 years represented a larger proportion of dengue cases, including more severe cases of dengue haemorrhagic fever and dengue shock syndrome, than children aged 0 to 4 years. By 2020, children aged 10 to 14 years were the most commonly represented age group among dengue patients (Fig. 4). Overall, the mean age of infected individuals increased significantly from 5.8 years (SE: 0.3) in 2002 to 9.1 years (SE: 0.4) in 2020 (slope: 0.20; SE: 0.0094; *P* -value: < 0.001; Fig. 5). This change corresponds to the increase in median age of the overall population as indicated in the 1998, 2008 and 2018 census surveys (online repository),^31^ reflecting Cambodia’s demographic transitions as a result of industrialization.

CI: confidence interval.

Note: The black line presents predictions of a generalized linear model fitted to dengue case age, excluding the effects of climate and seasonality.

### Trends in viral serotypes 

**Fig. 6:**
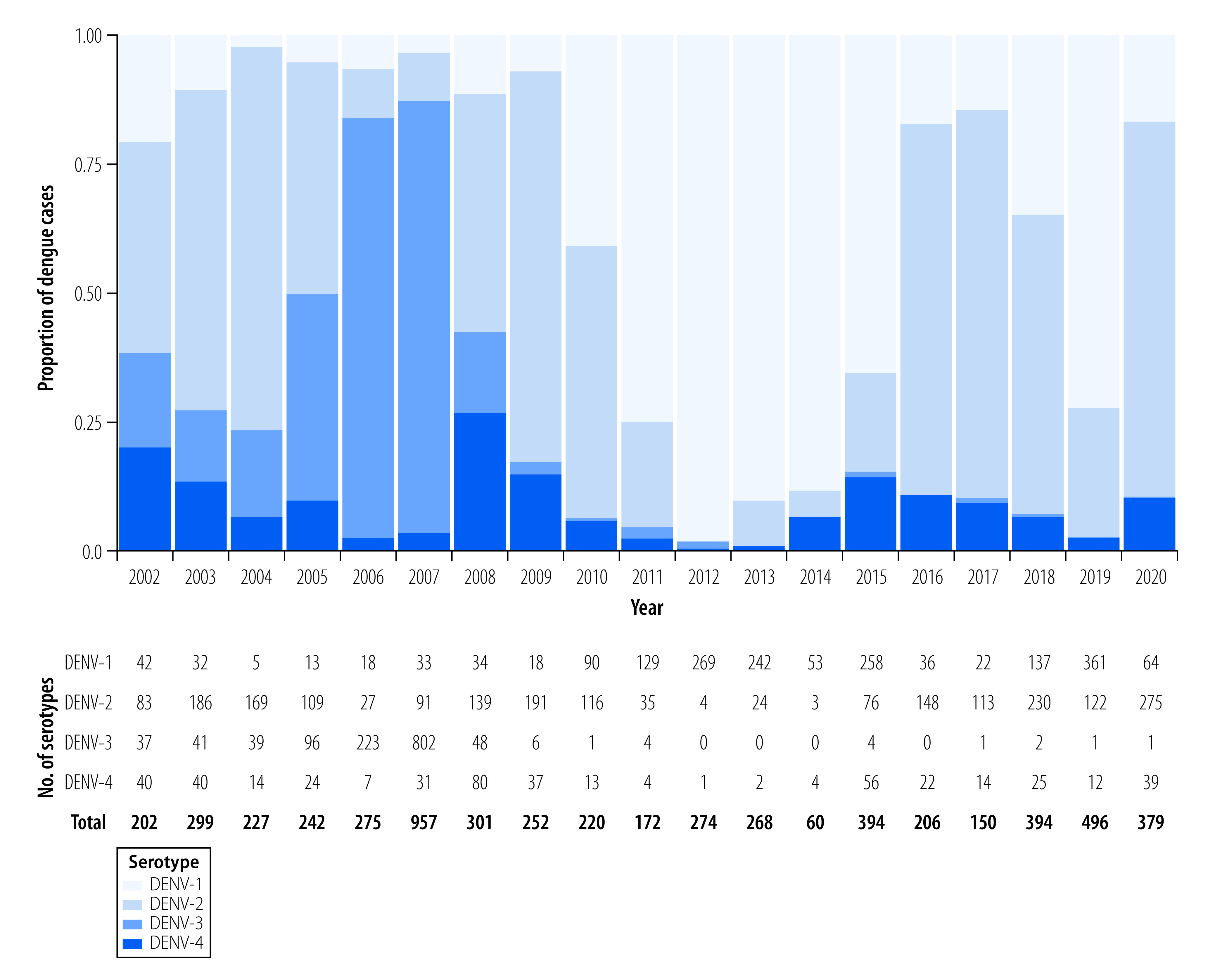
Distribution of dengue serotypes, Cambodia, 2002–2020

Note: The samples are from sentinel sites in 15 provinces.

The National Dengue Control Programme performs dengue serotype-specific RT–PCR on a monthly basis in a subset of samples from sentinel sites (Fig. 6). Before 2008, DENV-2 and DENV-3 serotypes were the predominant circulating serotypes, including an epidemic in 2007 driven by DENV-3 that preceded near-extinction of this serotype. Since 2008, DENV-1 and DENV-2 have dominated, with lower proportions of DENV-4 constituting approximately 10% of circulating variants. The 2012 epidemic was driven by DENV-1, most likely due to a genotype replacement of genotype IV to I that increased mosquito-virus transmission potential.^36^ During the large 2019 epidemic, DENV-1 and DENV-2 dominated; modelling studies are ongoing to investigate possible immunological, anthropological and ecological mechanisms of the outbreak. Studies in other countries variably attributed the high case numbers in 2019 to extreme weather events,^37^ human migration,^38^ and waning cross-protective immunity from prior arboviral outbreaks^39^ or with re-emergence of extinct genotypes.^37^

### Climate factors

Annual peaks in dengue cases occurred in June through August, approximately 3 months after peak temperatures and preceding peak precipitation by 1–2 months (online repository).^31^ Both precipitation and temperature demonstrated significant intra-annual seasonality but no significant change between 2002 and 2020 (online repository).^31^ These climate factors strongly predicted seasonality and annual dengue cases (online repository);^31^ however, increases in dengue incidence remained significant (*P*-value: < 0.001) even after adjustment for both factors. These results suggest that while climate changes contributed to rising dengue incidence in the last two decades, other factors may also be responsible for the increase. Additionally, the seasonal effects of temperature and precipitation reinforce the utility of including climate variables in epidemic forecasting systems. Notably, potential micro-effects at the village, district and/or province level cannot be evaluated in this analysis.^40^^,^^41^

### Vector control

The past two decades of development in Cambodia have led to changes in land use and, as a result, vector habitats. The World Bank estimates that forested area decreased by approximately 24% between 2002 to 2020, from 107 000 km^2^ (61% country land mass) to 81 000 km^2^ (46%), while the percentage of the population living in urban dwellings has increased from 19% (2 364 127/12 561 779) to 24% (3 973 287/16 396 860).^42^ How these changes have affected vector populations in Cambodia have yet to be studied: vector density surveys are not routinely performed in Cambodia; where data are available, larval and adult vector density does not correlate with measures of mosquito exposure.^43^

### Other *Aedes*-borne outbreaks

While autochthonous transmission occurred in Singapore during the 2015–2016 Zika disease outbreaks,^44^ only sporadic Zika virus cases were reported between 2007 and 2020 in Cambodia.^12^^,^^45^^,^^46^ Chikungunya virus re-emerged in Cambodia in 2011 with introduction of the east-central South African genotype,^47^ but burden was not routinely assessed in national surveillance and outbreaks may have gone undetected until a large epidemic in 2020.^12^^,^^48^ During this outbreak, the National Dengue Control Programme introduced arboviral differentiation RT–PCR in its sentinel surveillance programme. The 2020 outbreak consisted of 7014 suspected cases across 23 provinces; in 2021, a total of 1421 cases were reported across 15 provinces.^49^ To date, yellow fever has not been reported in Cambodia.

## Planning for the future

Despite expanded surveillance, enhanced data integration and improved disease management, national dengue surveillance in Cambodia continues to have several limitations including reliance on patient self-referral; predominant clinical syndrome-based identification of disease; exclusion of patients seeking care at private health-sector facilities; and limited integration with effective vector control efforts. In the 2021–2030 *National strategic plan on sustainable prevention and control of dengue and other Aedes-transmitted arboviral disease through a comprehensive integrated approach*,^49^ the National Dengue Control Programme identified several specific objectives (Table 1) to achieve three main targets by 2030: (i) reduce case fatality rates to goal of 0%; (ii) provide 100% detection and response to anticipated outbreaks; and (iii) reduce disease incidence by 50% (from 25 000 to 12 500 cases per year). 

**Table 1:** Specific objectives and proposed actions of 2021–2030 national strategic plan on sustainable prevention and control of dengue and other *Aedes-*transmitted arboviral diseases, Cambodia

Objective	Proposed steps	Additional considerations
Implementing integrated vector management	• Perform routine vector surveillance • Integrate entomological data in early warning systems • Deploy targeted use of insecticides	• Rising vector resistance to the larvicide temephos should prompt adoption of alternative methods of vector control
Improving environmental management	• Develop legislation to enforce sustainable waste management that is integral to vector control	• Changing land use and standards of living may lead to movement of the host-vector interface away from the home and into public spaces. Understanding where exposure occurs is needed to target the right environments
Strengthening early diagnosis	• Establish a national network of referral laboratories equipped with serologic and virologic testing capacity for arboviral disease diagnosis • Increase availability of point-of-care rapid diagnostic tests • Ensure widespread access to basic haematology tests to aid early recognition of complicated cases • Strengthen central oversight and quality assurance by the national dengue reference laboratory	• Advanced surveillance techniques introduced during the COVID-19 pandemic may help predict large outbreaks resulting from antigenic shifts
Enhancing clinical management	• Establish centres of excellence in high-risk areas to centralize management of complicated cases • Provide regular onboarding and refresher training on dengue management for clinicians • Perform regular root-cause analysis for dengue deaths to identify areas for improvement	Periodic active febrile and serologic surveys can help quantify true disease burden to inform control measures
Enhancing epidemic preparedness	• Collaborate with other federal agencies to enhance deployment of surge personnel and supplies during dengue outbreaks • Link existing early warning systems to climate data to allow detection of climate-driven increases in viral transmission	Periodic active febrile and serologic surveys can help quantify true disease burden to inform control measures
Reinforcing outbreak response	• Expand outbreak taskforces to include dedicated community dengue control teams led by village health support groups for community engagement, with a goal of monthly household visits during peak transmission seasons in districts with high caseloads	Deployment of pathogen-agnostic techniques in outbreaks may help identify novel viral genotypes or resembling pathogens

COVID-19: coronavirus disease 2019.

### Additional considerations

Dengue control relies upon close monitoring of each component in the host–vector–virus triad. Current surveillance in Cambodia centres around description of the host and virus, with limited focus on the vector. Integration of vector surveillance and control measures will provide a new, important dimension to the National Dengue Control Programme, with benefits to both containment of dengue and other *Aedes*-borne diseases. However, rising temephos resistance may require adoption of alternative methods of vector control.

Viral serotyping is available and performed on a subset of surveillance samples; widespread adoption of advanced serologic assays during the coronavirus disease 2019 (COVID-19) pandemic may help boost understanding and anticipation of unusual fluctuations in population susceptibility preceding large epidemics. Similarly, in-country access to pathogen-agnostic technologies such as metagenomic sequencing^50^ can be harnessed for future responses to outbreaks caused by a novel viral genotype or a look-alike pathogen. 

To develop effective interventions, improvements directed at better capture and characterization of at-risk populations are needed. Dengue has been classically described as a disease of the young, but recent trends in Cambodia and other countries may indicate the need for a paradigm shift.^51^ This transition to older age groups could indicate waning exposures to vectors, attributed to improved living conditions and sanitation. Movement of the host-vector interface away from the home and into public spaces, such as schools and workplaces, has important implications for where vector control measures are implemented.

In the clinical area, disease recognition in non-paediatric populations remains poor^12^ and may contribute to diagnostic delays. In addition, less familiarity with treating adult patients with distinct co-morbidities and risk profiles may contribute to higher morbidity and mortality in this population.^52^ While Cambodian treatment guidelines were updated in 2018 to inform management of adult patients with distinct co-morbidities and risk profiles, dedicated clinical studies in this age group along with educating providers and the public in symptom recognition and management will be important to ensure appropriate diagnosis and treatment. 

At a population level, accurate and timely capture of affected populations through continued passive surveillance combined with periodic febrile and serologic surveillance will continue to provide valuable insights to guide rollout of control measures.

## Conclusion

While the incidence of reported dengue cases in Cambodia increased between 2002 and 2020, the true burden of disease remains underestimated. Despite this, the National Dengue Control Programme has had notable successes reflected by reductions in cases of severe dengue and case fatality rates. For future interventions to reach susceptible populations at the appropriate scale, they will need to consider disease underestimation, shifting demographics, fluctuating viral serotypes, changing climate and land use, and novel emerging infectious threats.
